# The impact of third- or fourth-degree perineal tears on the second pregnancy: A cohort study of 182,445 Scottish women

**DOI:** 10.1371/journal.pone.0215180

**Published:** 2019-04-11

**Authors:** Andrea Mary Woolner, Dolapo Ayansina, Mairead Black, Sohinee Bhattacharya

**Affiliations:** 1 Aberdeen centre for Women’s Health Research, Institute of Applied Health Sciences, School of Medicine, Medical Sciences and Nutrition, University of Aberdeen, Aberdeen, United Kingdom; 2 Medical Statistics team, Institute of Applied Health Sciences, School of Medicine, Medical Sciences and Nutrition, University of Aberdeen, Aberdeen, United Kingdom; Azienda Unita Sanitaria Locale di Reggio Emilia, ITALY

## Abstract

This study aimed to investigate the reproductive impact of a third- or fourth-degree tear in primigravid women. A retrospective population-based cohort study was conducted using data from Scottish Morbidity Records (SMR02). Primigravid women with a vaginal birth in Scotland from 1997 until 2010 were included. Exposure was third- or fourth-degree tear in the first pregnancy. The second pregnancy rate, interpregnancy interval and third- or fourth-degree tear in a second pregnancy were the primary outcomes. A nested case-control study was used to determine factors associated with repeat third- or fourth-degree tears in a second vaginal birth. Cox regression analysis and logistic regression were used to look for associations. Initial third- or fourth-degree tear occurred in 2.8% women (5174/182445). The percentage of third- or fourth-degree tears in first vaginal births increased from 1% in 1997 to 4.9% in 2010. There was no difference in having a second pregnancy (adjusted Odds Ratio (aOR) 0.98 (99%CI 0.89–1.09)) or the median interpregnancy interval to second pregnancy (adjusted Hazard Ratio (aHR) 1.01 (99%CI 0.95–1.08)) after an initial third- or fourth-degree tear. Women were over four times more likely to have a repeat injury in a subsequent vaginal birth (n = 149/333, aOR 4.68 (99% 3.52–6.23)) and were significantly more likely to have an elective caesarean section in their second pregnancy (n = 887/3333, 26.6%; 12.75 (11.29–14.40)). Increased maternal age and birthweight ≥4500g were risk factors for repeat injury. Third- and fourth-degree tears are increasing in Scotland. Women do not delay or avoid childbirth after initial third- or fourth-degree tear. However, women are more likely to have a repeat third- or fourth-degree tear or an elective caesarean section in the second pregnancy. Strategies to prevent third- or fourth-degree tears are needed.

## Introduction

Third- and fourth-degree tears are injuries which can occur to the perineum during vaginal birth. A third degree tear extends through the anal sphincter muscle complex.[1–3] A fourth degree tear extends into the rectal mucosa.[1–3] Together they are often known as OASIS (Obstetric Anal Sphincter InjurieS).[1–3] Incidence of such tears appears to be rising with 5.9% of first pregnancies affected in England and Wales in 2012.[4] Both third and fourth degree tears can cause distressing symptoms such as faecal incontinence[3,5–8] and sexual dysfunction.[6,9] Childbirth following such a tear risks worsening symptoms[5,7,8] and repeat OASIS injury in a subsequent pregnancy.[4,10–18] However, evidence of the effect an initial third or fourth degree tear has on subsequent pregnancy and birth, including the risk of repeat injury, is limited. Consequently, it is difficult to advise women on future birth options. Women are offered either vaginal birth or elective caesarean section after such tears with little evidence to support either decision.[3] Women may delay or avoid future childbirth due to fears over repeat injury,[6] but evidence on time to next pregnancy is sparse. More information is needed so that women can make informed decisions about their second birth following an initial third- or fourth-degree tear.

This observational study is the first to generate precise risk information on pregnancy following third- or fourth-degree tears in Scottish women, using national data. First, we aimed to investigate the odds of having a second birth and the interpregnancy interval to second birth for women with and without an initial third- or fourth-degree tear. Second, we aimed to determine the odds of a repeat third- or fourth-degree tear in a second pregnancy. Finally, we aimed to determine any risk factors associated with repeat third- or fourth-degree tear in a second pregnancy.

## Methods

### Study design and conduct

This was a retrospective cohort study with a nested case-control study. This study is reported in accordance with the STROBE statement for observational studies.[19] Data was obtained from Information Services Division Scotland (ISD; http://www.isdscotland.org) for all women with a first vaginal singleton birth in Scotland between 1997 and 2010. This included all women who gave birth vaginally in a National Health Service (NHS) Scottish hospital and whose medical records were routinely collected as part of the Scottish Morbidity Records (SMR02). The SMR02 dataset holds maternity data for women in Scotland from 1980. Eligible women were identified by the electronic Data Research and Innovation Service (eDRIS, a part of ISD Scotland). Women were included if they had a live born baby in their first pregnancy. Vaginal birth included spontaneous and operative vaginal birth. Women were included if they had information recorded within SMR02 data of their perineal or vaginal trauma at time of birth. Women who had twins or higher multiples delivered vaginally in the first pregnancy were excluded. Women were followed up from 1998 to 2015 to identify any second pregnancies.

### Retrospective cohort study

Exposure was defined as a third- or fourth-degree tear in the first pregnancy. Women without a third- or fourth-degree tear in their first pregnancy were the unexposed group. The unexposed group included women with no perineal tear, first or second-degree perineal tears, other vaginal lacerations or unspecified tears. A second birth, the interpregnancy interval and a third- and fourth-degree tear in the second pregnancy were the primary outcomes. Secondary outcomes included mode of delivery, gestation at birth and episiotomy use in the second pregnancy.

Potential confounders adjusted for in the multivariate analysis to investigate second pregnancy rate and interpregnancy interval were age (in years); body mass index (BMI); socioeconomic deprivation (Carstairs 2011 deciles[20,21]: 1–5 most deprived, 6–10 least deprived); and smoking status (non-smoker, smoker, ex-smoker). These covariates were all recorded in the first pregnancy.

Subsequently the odds of having a third- or fourth-degree tear at the time of the second pregnancy if a woman had an initial third- or fourth-degree tear was investigated. The odds of a third of fourth degree tear in a second pregnancy was calculated and adjusted for potential confounding factors at the second pregnancy level. Potential confounders included in the multivariate analyses were: age (in years); body mass index (BMI); socioeconomic deprivation (Carstairs 2011 deciles[20,21]: 1–5 most deprived, 6–10 least deprived); smoking status (non-smoker, smoker, ex-smoker); diabetes (yes/no, including pre-existing and gestational); mode of delivery (spontaneous vaginal delivery (SVD), non-rotational forceps, ventouse (any), rotational forceps, breech vaginal, elective caesarean section and emergency caesarean section; birthweight of baby (<4500g, ≥4500g); gestation at birth (weeks); episiotomy (yes/no); induction of labour (none, ARM only, ARM and pharmacological methods); and position at delivery (OA, OP, OT, Other). Using confounders at the second pregnancy level was felt to be clinically relevant, as women would want to know their chance of having another third- or fourth-degree tear with their current risk factors in a second pregnancy according to their exposure of a previous third- or fourth-degree tear.

A sample size of 3602 women with the exposure of an initial third- or fourth-degree tear was estimated and an unexposed cohort of 183, 492 women. Repeat third or fourth degree tears have a published frequency of 4.0–5.6 per 100 women.[10,11] A third or fourth degree tear in a second pregnancy with no history of such tear has a published frequency of 0.6–0.8 per 100 women.[10,11] Based on these figures and using a 2-sided continuity corrected chi-squared test and a significance level of 0.01, our study was estimated to have 99% power to detect a 5% increase in odds of a third or fourth degree tear in a second pregnancy between the exposed and unexposed groups.

### Nested case-control study

A nested case-control was carried out to look for risk factors associated with repeat third- or fourth-degree tears in a second pregnancy. All women who had a third- or fourth-degree tear in the first pregnancy and who had a second vaginal birth were included. Cases were women with a repeat third- or fourth-degree tear in their second pregnancies. Controls were women who did not have a repeat third- or fourth-degree tear in their second pregnancy. Where tear in 2^nd^ pregnancy was “unspecified” these women were included as controls. Potential confounders included in multivariate analyses were: age (in years); body mass index (BMI); socioeconomic deprivation (Carstairs 2011 deciles[20,21]: 1–5 most deprived, 6–10 least deprived); smoking status (non-smoker, smoker, ex-smoker); diabetes (yes/no, including pre-existing and gestational); mode of delivery (spontaneous vaginal delivery (SVD), non-rotational forceps, ventouse (any), rotational forceps, breech vaginal, elective caesarean section and emergency caesarean section); birthweight of baby (<4500g, ≥4500g); gestation at birth (weeks); episiotomy (yes/no); induction of labour (none, ARM only, ARM and pharmacological methods); and position at delivery (OA, OP, OT, Other). The covariates were included at the second pregnancy level.

### Fourth degree tears

A subgroup analysis included women who had a fourth-degree tear in the first pregnancy as the exposed group. Women with initial fourth degree tears were compared to all other women (unexposed group). The odds of having a second pregnancy after an initial fourth degree tear were calculated and adjusted for age at first birth, BMI, smoking and deprivation category.

### Data confidentiality

Approval (Ref: 1516–0566) to conduct this study was obtained from the Public Benefit and Privacy Panel (PBPP) which is part of NHS Services Scotland. PBPP are responsible for approving access to Scottish national data. Following PBPP approval further ethical approval was not required. An anonymised dataset was provided to researchers which was only available within the national electronic Data Research and Innovation Service (eDRIS) Safe Haven, part of Information Services Scotland (http://www.isdscotland.org/Products-and-Services/eDRIS/). All data was anonymised before being made available to researchers within the Safe Haven. All researchers with access to the national Safe Haven underwent formal training in research data confidentiality. Data was not stored or linked in any way.

### Statistical analysis

All data were stored and analysed using SPSS software (*IBM Corp*. *Released 2016*. *IBM SPSS Statistics for Windows*, *Version 22*.*0*. *Armonk*, *NY*: *IBM Corp*.). Univariate and multivariate analyses were performed. A statistical significance level of 0.01 was used in all analyses given the large dataset used.

Binary logistic regression was used to determine the odds of having a second pregnancy in the exposed group compared to unexposed. Both crude and adjusted Odds Ratios (aORs) with 99% confidence intervals (CI) were calculated. Kaplan-Meier curves of time to second pregnancy (interpregnancy interval) were constructed and compared using the log rank test for women with and without an initial third- or fourth-degree tear. Women were censored at the point of last data collection (31^st^ December 2015). Cox’s regression analysis was used to calculate crude and adjusted hazard ratios (aHR) with 99% CI for second pregnancies. Binary logistic regression was used to determine the odds of having a third- or fourth-degree tear among women with a second pregnancy between those with an initial third- or fourth-degree tear and those without such a previous tear.

In the nested case-control study, binary logistic regression was used to look for associations in women with initial third- or fourth-degree tear who also sustained a repeat third- or fourth-degree tear compared to women with a first but not a second (repeat) third or fourth degree. Both crude and adjusted Odds Ratios (aORs) with 99% confidence intervals (CI) were calculated.

### Missing data

Complete case analysis was carried out and presented. Where more than 5% of covariate data was missing a sensitivity analysis was carried out. We planned to include ethnicity as a potential confounder, however as ethnicity was missing in >60% and as it could be assumed that the Scottish population would be predominantly a Caucasian population, ethnicity was not included as a covariate.

## Results

**Fig 1:**
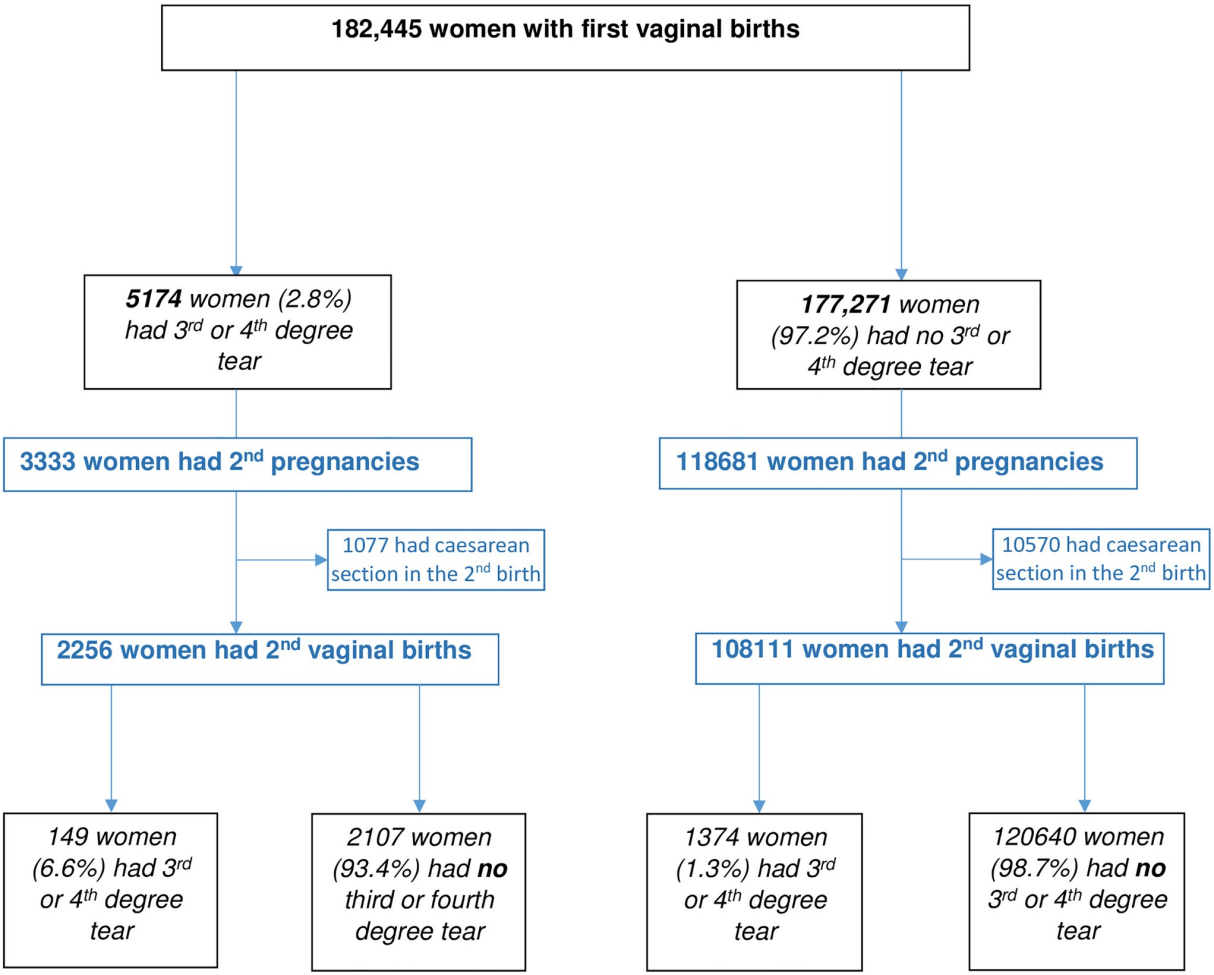
Flowchart of women included with their first birth in Scotland between 1997 and 2010.

**Fig 2:**
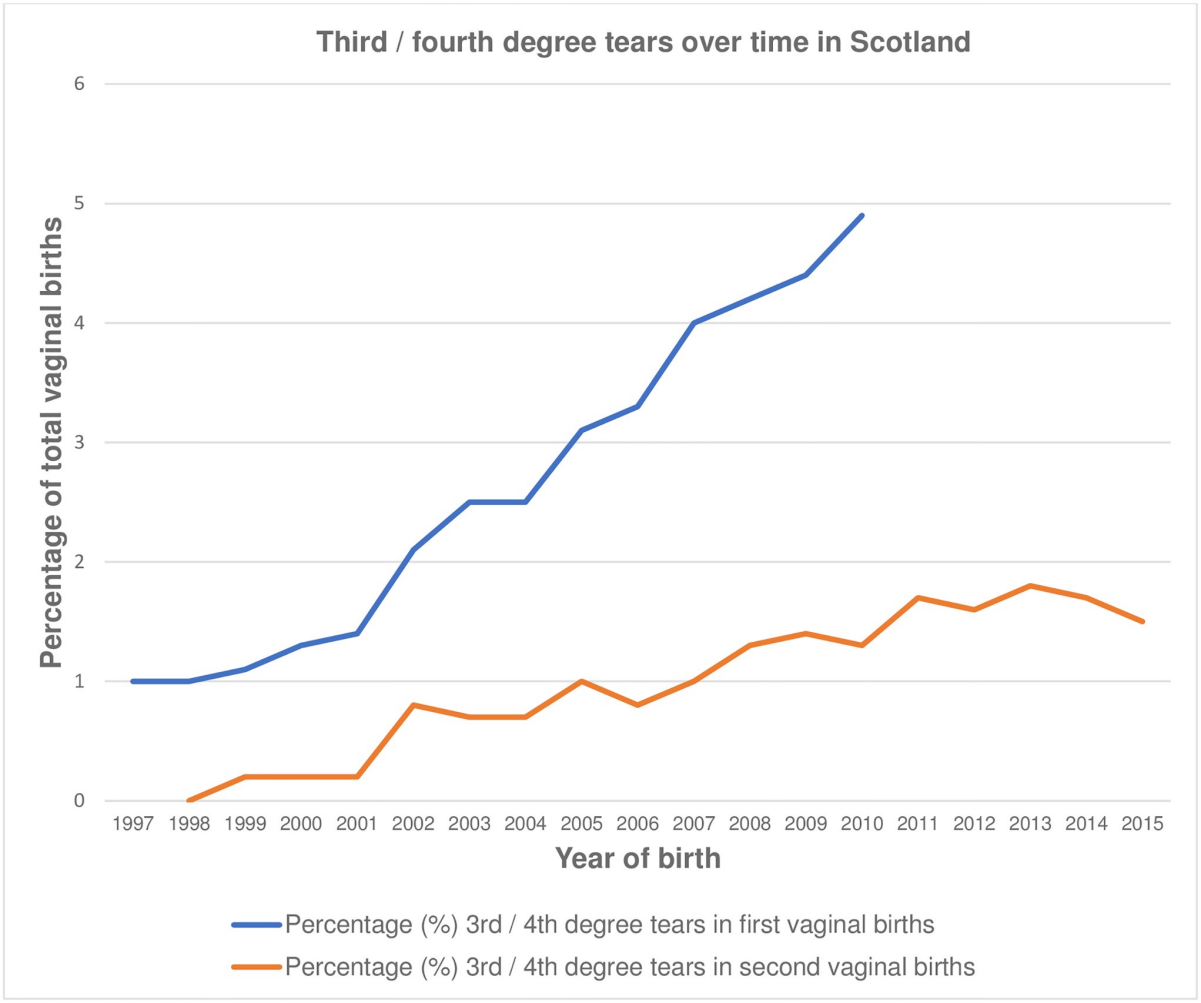
Third- or fourth-degree tears in first and second vaginal births over time in Scotland 1997–2015.

The study included 182,445 women with first vaginal births in Scotland between 1997 and 2010. The population sample is illustrated in Fig 1. 5174 (2.8%) of women had a third- or fourth-degree tear in their first pregnancy. 131015 (71.8%) women had a spontaneous vaginal birth whereas 3541 (19.4%) had a forceps delivery and 15347 (8.4%) had a ventouse delivery for their first vaginal birth. Second pregnancies were recorded between 1998 and 2015 in 122,014 women. Fig 2 shows the percentage of third- and fourth-degree tears over time for all women with first and second vaginal births in Scotland from 1997–2015. Over time the percentage of first vaginal births which resulted in a third- or fourth-degree tear has increased from 1% in 1997 to 4.9% in 2010. Similarly, the rate of third- or fourth-degree tears in second vaginal births is increasing, with 0.2% in 1999 to 1.5% in 2015, though the trajectory is less marked. Table 1 shows the baseline characteristics of the women in their first pregnancy according to exposure status. Women with an initial third- or fourth-degree tear were more likely to be older. However, there was no difference in BMI or socioeconomic deprivation between the exposed and unexposed groups. Notably, women without an initial third- or fourth-degree tear were more likely to be smokers. Table 1 also demonstrates the proportion of women who had a second birth and the odds of having a second birth according to tear status in the first vaginal birth. Women with an initial third- or fourth-degree tear were less likely to have a second pregnancy recorded with a difference of 2.5% (crude OR 0.89 (99% CI 0.83–0.96). However, this was not statistically significant when adjusted for potential confounders (aOR 0.98 (99%CI 0.89–1.09)).

**Table 1:** First pregnancy characteristics for women according to their exposure status.

First pregnancy characteristic	Initial 3^rd^ or 4^th^ degree tear n (%) (N = 5174)	No 3^rd^ or 4^th^ degree tear n (%) (N = 177271)	Unadjusted OR (99%CI)	Adjusted OR^a^ (99%CI)
**Second pregnancy**				
**Yes**	3333 (64.4)	118681 (66.9)	0.89 (0.83–0.96)	0.98 (0.89–1.09)
**No**	1841 (35.6)	58590 (33.1)		
**Age at delivery**				
***Missing = 2***				
**Mean (SD)**	27.6 (5.6)	25.9 (6.0)	1.05 (1.04–1.06)	1.04 (1.03–1.05)
**Body mass index**				
**Missing = 106231**				
**Mean (SD)**	25.0 (4.8)	25.0 (5.0)	1.00 (0.99–1.01)	1.00 (0.99–1.01)
**Deprivation**				
**Missing = 529**				
**Most deprived**	2765 (53.6)	102433 (58.0)	0.84 (0.78–0.90)	0.97 (0.88–1.07)
**Least deprived**	2395 (46.4)	74323 (42.0)	1	1
**Smoking status**				
**Missing = 12763**				
**Non smoker**	3506 (73.5)	105038 (63.7)	1	1
**Smoker**	671 (14.1)	40695 (24.7)	1.79 (1.47–2.18)	0.65 (0.56–0.75)
**Ex-smoker**	590 (12.4)	19182 (11.6)	1.54 (1.20–1.97)	0.96 (0.84–1.11)

^a^Multivariate analyses adjusted for age at delivery, BMI, deprivation, smoking (all in the first pregnancy)

Missing data not included in the proportions presented

There was no difference in interpregnancy interval for women with an initial third- or fourth-degree tear (median time to second pregnancy was 181 weeks) compared to women without an initial such tear (median time to second pregnancy = 202 weeks; log rank test p = 0.542). Using Cox regression analysis, the unadjusted HR 1.01 (99%CI 0.97–1.06) and adjusted HR 1.01 (99%CI 0.95–1.07) also show no statistically significant difference for rates of second pregnancy between the exposed and unexposed groups. Age (in years); body mass index (BMI); socioeconomic deprivation (Carstairs 2011 deciles[20,21,21]: 1–5 most deprived, 6–10 least deprived); and smoking status (non-smoker, smoker, ex-smoker) all from the first pregnancy were included as covariates in this adjusted analysis.

Table 2 shows the demographic and pregnancy outcomes for women who had second pregnancies according to their initial tear status. 1374 (1.1%) women had third- or fourth-degree tears in their second pregnancy (of all women who had second pregnancies, N = 122,014). Of the women who had an initial third- or fourth-degree tear and who had a second pregnancy (N = 3333), 149 (4.5%) had a repeat third- or fourth-degree tear. After exclusion of women who delivered their second baby by caesarean section (either elective or emergency) the percentage of repeat third or fourth degree tears in the second pregnancy for women with an initial third or fourth degree tear was 6.6% (149/2254, Fig 1), with risk of such a tear in a second pregnancy being significantly increased compared to the control group {crude OR 6.18 (99%CI 4.91–7.78); aOR 4.68 (99%CI 3.52–6.23)}. 1225 (1.0%) women had their first 3^rd^ or 4^th^ degree tear in the second pregnancy. Only 64.1% women with an initial third- or fourth-degree tear had a spontaneous vaginal delivery (SVD) in the second pregnancy (compared to 86.8% of controls). Table 3 shows the comparison of second pregnancy outcomes for women with and without an initial third- or fourth-degree tear. Women with a third- or fourth-degree tear in the first pregnancy were over twelve times more likely to have an elective caesarean section {(n = 887/3333, 36.6%; aOR 12.75 (99%CI 11.29–14.40)} and twice as likely to have an emergency caesarean section {n = 190, 5.7%; aOR 2.42 (99%CI 2.01–2.93)}. Episiotomy use for the second birth was over three times more likely in women with a previous third- or fourth-degree tear (aOR 3.74 (99%CI 3.25–4.32). Women with an initial third- or fourth-degree tear were less likely to be induced in their second pregnancy. There was no significant difference in weight of second baby or position of baby at birth. A statistically but not clinically significant difference was noted between groups for gestation at second birth. Specifically, the difference in gestation was 0.1 weeks which is not a clinically significant period of time. Women with an initial third- or fourth-degree tear were older at delivery in the second pregnancy compared to women without an initial injury. This could be explained by there being no statistically significant difference in interpregnancy interval for women with and without an initial third- or fourth-degree tear, as women with a third- or fourth-degree tear in the first pregnancy were notably significantly older at first vaginal birth than those without.

**Table 2:** Univariate comparison of demographic and pregnancy characteristics for women who had second pregnancies.

Second pregnancy characteristic	Initial 3^rd^ or 4^th^ degree tear n (%) (N = 3333)	No prior 3^rd^ or 4^th^ degree tear n (%) (N = 118681)	P-value
**Third/fourth degree tear Yes**	149 (4.5)	1225 (1.0)	
**No**	3184 (95.5)	117456 (99.0)	<0.01*
**Age**			
**Missing = 1**			
**Mean (SD)**	30.5 (5.2)	29.0 (5.5)	<0.01*
**Smoking**			
**Missing = 6584 (5.4%)**			
**Non smoker**	2480 (78.1)	76880 (68.5)	
**Smoker**	380 (12.0)	24113 (21.5)	<0.01*
**Ex-smoker**	317 (10.0)	11260 (10.0)	0.014
**Deprivation**			
**Missing = 302 (0.2)**			
**Most deprived (1–5)**	1648 (49.6)	64917 (54.8)	<0.01*
**Least deprived (6–10)**	1675 (50.4)	53472 (45.2)	
**Diabetes**			
**Missing = 19873 (16.3%)**			
**Yes**	36 (1.2)	1261 (1.3)	0.684
**No**	2994 (98.8)	97850 (98.7)	
**BMI**			
**Missing = 44646 (36.6%)**			
**Mean (SD)**	25.9 (5.2)	26.0 (5.4)	0.039
**Mode of delivery**			
**Missing = 24 (0.0%)**			
**SVD**	2134 (64.1)	103019 (86.8)	
**Forceps (non rot)**	61 (1.8)	2453 (2.1)	0.165
**Ventouse (all)**	33 (1.0)	1660 (1.4)	0.956
**Forceps (rotational)**	15 (0.4)	559 (0.5)	0.195
**Breech vaginal**	11 (0.3)	398 (0.3)	0.927
**Elective CS**	887 (26.6)	4416 (3.7)	<0.01*
**Emergency CS**	190 (5.7)	6154 (5.2)	<0.01*
**Weight of baby (grams)**			
**Missing = 87 (0.1%)**			
**<4500g**	3233 (97.1)	115520 (97.4)	0.213
**≥4500g**	98 (2.9)	3076 (2.6)	
**Gestation at birth (weeks)**			
**Missing = 119 (0.1)**			
**Mean (SD)**			
	39.4 (2.8)	39.5 (2.5)	0.07
**Episiotomy**			
**Missing = 1812 (1.5%)**			
**Yes**	486 (15.0)	8344 (7.4)	<0.01*
**No**	2758 (85.0)	104001 (92.6)	
**Induction of labour**			
**Missing = 447 (0.4)**			
**None**	2821 (84.8)	90722 (76.7)	
**ARM only**	101 (3.0)	5524 (4.7)	<0.01*
**ARM + pharmacological**	406 (12.2)	21993 (18.6)	<0.01*
**Position at delivery**			
**Missing = 7228 (5.9)**			
**OA**	2690 (88.4)	101144 (90.5)	
**OP**	102 (3.4)	3472 (3.1)	0.331
**OT**	118 (3.9)	3069 (2.7)	<0.01*
**Other**	134 (4.4)	4057 (3.6)	0.016

Missing data not included in the proportions presented.

*denotes statistical significance.

**Table 3:** Crude and adjusted Odds Ratios (99% CI) for second pregnancy outcomes for women with and without an initial third- or fourth-degree tear.

Second pregnancy characteristic	Unadjusted OR (99%CI)	Adjusted OR(99%CI)^a^
**Third/fourth degree tear**		
**Yes**	4.49 (3.57–5.64)	4.84 (3.64–6.46)
**No**	1	1
**Age**		
**(Missing = 1)**		
**Mean (SD)**	1.05 (1.04–1.06)	1.02 (1.00–1.03)
**Smoking**		
**Missing = 6584 (5.4%)**		
**Non smoker**	1	1
**Smoker**	0.49 (0.42–0.56)	0.67 (0.58–0.77)
**Ex-smoker**	0.87 (0.75–1.02)	0.83 (0.72–0.96)
**Deprivation**		
**Missing = 302 (0.2)**		
**Most deprived (1–5)**	0.81 (0.74–0.89)	0.90 (0.82–0.98)
**Least deprived (6–10)**	1	1
**Diabetes**		
**Missing = 19873 (16.3%)**		
**Yes**	0.93 (0.60–1.45)	0.79 (0.53–1.17)
**No**	1	1
**BMI**		
**Missing = 44646 (36.6%)**		
**Mean (SD)**	1.00 (0.99–1.01)	0.99 (0.98–1.00)
**Mode of delivery**		
**Missing = 24 (0.0%)**		
**SVD**	1	1
**Forceps (non rot)**	1.20 (0.86–1.68)	0.36 (0.25–0.51)
**Ventouse (all)**	1.01 (0.68–1.51)	0.65 (0.44–0.96)
**Forceps (rotational)**	1.41 (0.71–2.77)	0.45 (0.25–0.82)
**Breech vaginal**	1.10 (0.08–14.85)	0.63 (0.08–4.65)
**Elective CS**	9.70 (8.68–10.83)	12.75 (11.29–14.40)
**Emergency CS**	1.49 (1.22–1.82)	2.42 (2.01–2.93)
**Weight of baby (grams)**		
**Missing = 87 (0.1%)**		
**<4500g**	1	1
**≥4500g**	1.14 (0.87–1.49)	0.84 (0.59–1.18)
**Gestation at birth (weeks)**		
**Missing = 119 (0.1)**		
**Mean (SD)**	0.99 (0.97–1.01)	1.01 (1.00–1.03)
**Episiotomy**		
**Missing = 1812 (1.5%)**		
**Yes**	2.20 (1.93–2.50)	3.74 (3.25–4.32)
**No**	1	1
**Induction of labour**		
**Missing = 447 (0.4)**		
**None**	1	1
**ARM only**	0.59 (0.45–0.77)	0.73 (0.56–0.93)
**ARM + pharmacological methods**	0.59 (0.52–0.68)	0.78 (0.68–0.89)
**Position at delivery**		
**Missing = 7228 (5.9)**		
**OA**	1	1
**OP**	1.11 (0.85–1.44)	0.88 (0.64–1.23)
**OT**	1.24 (0.99–1.57)	0.97 (0.72–1.31)
**Other**		0.30 (0.22–0.41)

^a^Covariates included in the multivariate model were third- or fourth-degree tear in 2^nd^ pregnancy, age, smoking, deprivation, diabetes, BMI, mode of delivery, weight of baby, gestation, episiotomy, induction of labour and position at delivery (all in second pregnancy)

Missing data not included in the proportions presented.

### Risk factors for repeat third- or fourth-degree tear

The results of the nested case-control study for all women with a previous third- or fourth-degree tear (n = 2254) who had a second vaginal birth are shown in Table 4. Women with repeat third- or fourth-degree tears in the second pregnancy appeared to be older, however there was no association seen with smoking, deprivation, diabetes, BMI, mode of delivery, gestation at birth, episiotomy use, induction of labour or position of baby’s head at birth. However, birthweight ≥4500g was significantly associated with a repeat third- or fourth-degree tear (aOR 3.27 (99%CI 1.11–9.60)).

**Table 4:** Demographic and pregnancy characteristics for all women with an initial third- or fourth-degree tear who had a second vaginal birth (nested case-control study).

Second pregnancy characteristic	Repeat 3^rd^ / 4^th^ degree tear n (%) (N = 149)	No repeat 3^rd^ / 4^th^ degree tear n (%) (N = 2105)	P-value	Unadjusted OR (99%CI)	Adjusted OR (99%CI)^a^
**Age**					
**Missing = 0**					
**Mean (SD)**	31.5 (4.6)	30.1 (5.3)	0.002*	1.05 (1.01–1.10)	1.06 (1.00–1.12)
**Smoking**					
**Missing = 104 (4.6)**					
**Non smoker**	124 (86.7)	1535 (76.5)		1	1
**Smoker**	7 (4.9)	260 (12.4)	0.005*	0.33 (0.12–0.92)	0.61 (0.21–1.80)
**Ex-smoker**	12 (8.4)	212 (10.1)	0.253	0.70 (0.31–1.56)	0.54 (0.19–1.54)
**Deprivation**					
**Missing = 8 (0.4)**					
**Most deprived (1–5)**	80 (53.7)	1010 (48.2)	0.661	1.08 (0.70–1.67)	1.20 (0.69–2.09)
**Least deprived (6–10)**	69 (46.3)	1087 (51.6)		1	1
**Diabetes**					
**Missing = 223 (9.9)**					
**Yes**	2 9(1.4)	1872 (99.0)	0.634	1.43 (0.21–9.82)	1.05 (0.69–15.96)
**No**	138 (98.6)	19 (1.0)		1	1
**BMI**					
**Missing = 577 (25.6)**					
**Median (IQR)**	24.9 (6.4)	24.7 (6.4)	0.789	1.00 (0.95–1.05)	0.99 (0.93–1.05)
**Mode of delivery**					
**Missing = 0**					
**SVD**	145 (97.3)	1989 (94.5)	0.602	1	1
**Forceps**	4 (2.7)	72 (3.4)	0.998	0.76 (0.20–2.92)	0.55 (0.07–4.15)
**Ventouse**	0	33 (1.6)	0.999	—	—
**Vaginal breech**	0	11 (0.5)		—	—
**Weight of baby (grams)**					
**Missing = 2 (0.1)**					
**<4500g**					
**≥4500g**	139 (93.3)	2045 (97.2)	0.008*	1	1
	10 (6.7)	58 (2.8)		2.54 (1.02–6.30)	3.27 (1.11–9.60)
**Gestation at birth**					
**(weeks)**					
**Missing = 0**					
**Median (IQR)**	40 (1.0)	40 (2.0)	0.335	1.02 (0.97–1.07)	1.01 (0.95–1.08)
**Episiotomy**					
**Missing = 59 (2.6)**					
**Yes**	27 (18.2)	456 (22.3)	0.254	0.78 (0.44–1.37)	0.88 (0.43–1.84)
**No**	121 (81.8)	1591 (77.7)		1	1
**Induction of labour**					
**Missing = 5 (0.2)**					
**None**					
**ARM only**	119 (79.9)	1664 (79.2)		1	1
**ARM +PG**	10 (6.7)	82 (3.9)	0.125	1.71 (0.70–4.18)	1.81 (0.60–5.44)
	20 (13.4)	354 (16.9)	0.343	0.79 (0.42–1.50)	0.68 (0.29–1.58)
**Position at delivery**					
**Missing = 139 (6.2)**					
**OA**	125 (90.6)	1831 (87.0)		1	1
**OP**	7 (5.1)	67 (3.2)	0.297	1.53 (0.54–4.38)	2.06 (0.62–6.82)
**OT**	5 (3.6)	49 (2.3)	0.401	1.50 (0.44–5.13)	0.84 (0.06–12.22)
**Other**	1 (0.7)	30 (1.4)	0.482	0.49 (0.04–6.77)	—

^a^Covariates included in the multivariate model were age, smoking, deprivation, diabetes, BMI, mode of delivery, weight of baby, gestation, episiotomy, induction of labour and position at delivery (all in second pregnancy)

Missing data not included in the proportions presented.

*denotes statistical significance.

### Fourth degree tears

Of all third- and fourth-degree tears in first vaginal births, 260 (5.0%) were fourth degree tears. This equates to 0.1% (260/182445) of all primigravid women with first vaginal births. There was no difference in the odds of having a second birth for women with an initial fourth degree tear compared to women with any other type of tear or no tear in the first pregnancy (aOR 1.01 (99%CI 0.64–1.58)). When women with initial fourth degree tears were compared to all other women, there was no significant difference in the interpregnancy interval in crude or adjusted analyses ((crude HR 0.91 (99%CI 0.75–1.12); aHR 0.98 (99%CI 0.76–1.28)). Of the women who had a repeat third- or fourth-degree tear, 6.7% (10/149) had a 4^th^ degree tear in the second pregnancy.

## Discussion

### Main findings

Third- and fourth-degree tears are becoming more prevalent in first and second vaginal births in Scotland. Women do not appear to delay future childbirth following an initial third- or fourth-degree tear. There was however a 2.5% reduction in second pregnancies for women with an initial third- or fourth-degree tear over this time period but this was not statistically significant after adjustment for confounding factors. Having an initial fourth degree tear does not influence time to a second pregnancy. Women with an initial third or fourth degree were twelve times more likely to have an elective caesarean section in a second pregnancy. Women with an initial third- or fourth-degree tear who opted for a vaginal birth in the second pregnancy were significantly more likely to have a repeat third- or fourth-degree tear. Of all repeat third- or fourth-degree tears, 6.7% were fourth degree tears. Episiotomy use appears to increase in the second vaginal birth for women with prior third- or fourth-degree tear. Increasing maternal age and a baby’s birthweight of ≥4500g were associated with repeat third- or fourth-degree tears.

### Strengths and limitations

This is the first study to determine precise risk estimates for Scottish women with third- or fourth-degree tears in their first pregnancy. This was a large sample of routinely collected registry-based hospital data thereby eliminating the risk of recall bias. The SMR02 data is a high-quality dataset with information coded from each maternity unit in Scotland providing a large population sample. However, our sample size could be affected due to the time period included as women whose first birth was at the latter end of our inclusion period may not yet have delivered their second babies. This could have been improved by including a larger cohort over a longer time period. However, using a historical cohort is likely to be unhelpful when studying third- and fourth-degree tears as tear classifications have changed over time. Furthermore, given the small numbers of women who had a second vaginal birth after an initial third or fourth degree tear it is possible this study is underpowered to detect significant differences in the risk factors studied in the nested case-control study.

We were able to include multiple confounding factors in the multivariate analyses in the retrospective cohort study at the first or second pregnancy level where appropriate due to the level of data collected within the SMR02 dataset. This increases the reliability of the associations found and the strength of our results. We used a stringent 0.01 level of significance to reduce the risk of spurious positive associations due to the multiple analyses performed within such a large dataset. Given the association with Asian ethnicity and third or fourth degree tears[4] it would have been preferable to include ethnicity as a covariate in the multivariate analyses. In this study we were unable to differentiate between grades of third-degree tear (3A, B, C) or types of fourth degree tear (buttonhole versus rectal mucosal injuries secondary to full thickness injury to the external anal sphincter and internal anal sphincter). Given that tears involving the internal anal sphincter or rectal mucosa are thought to carry a worse prognosis,[22] this is a limitation to our results. Furthermore, women with a more severe third-degree tear or a fourth-degree tear may be more likely to have subsequent Caesarean section for their second birth.

Women with an initial third- or fourth-degree tear were more likely to have an elective caesarean section in their second pregnancy. However, we did not have access to the indication for the caesarean section so it is possible that this was not related to their initial third- or fourth-degree tear. Given the stark difference between the two groups it seems reasonable to assume that the history of initial third or fourth degree is likely to be a contributory factor to the decision for subsequent mode of delivery at least for a proportion of these women. However, there is no certainty, therefore this is a limitation of our results.

### Interpretation

There is an increasing prevalence of third and fourth degree tears in our sample which mimics that presented in other similar populations.[4,23,24] This may be secondary to improving classification and recognition of such tears,[4,23,24] however the gradual trend seen in our sample would suggest this increase is not secondary to a significant change in education. Instead this could be due to an increasing prevalence of predisposing risk factors such as bigger babies or the trend for women to be older at first pregnancy. In this Scottish sample, we found a slightly lower prevalence of third and fourth degree tears in the first pregnancy (ranging from 1% in 1997 to 4.9% in 2010%) than that reported for England and Wales of 5.9% in 2012.[4] This may be due to differences in the population, maternal risk factors, obstetric practice or training. The overall rate of repeat tears in women with second vaginal births was again slightly lower (6.6%) than reported from national English hospital data where the rate of repeat third or fourth degree tears was 7.2%[25].

Our results disagree with previous research which suggests women with severe perineal tears were less likely to have another baby.[12,26] Though an observational study in Finland also found there was no difference in subsequent delivery rate after an initial third or fourth degree tear.[10] This could be due to greater acceptance of the option of planned caesarean section following initial third or fourth degree tear in Scotland.

Several studies suggest there is a significant risk of repeat third or fourth degree tear for women with an initial injury.[10–16,18,25] However, it is impossible to provide women with a realistic estimate from our population of the risk of repeat injury, nor similar populations where caesarean section is offered for second births, as many women appear to have subsequent planned caesarean sections. We estimate that women are four times more likely to have a repeat injury but it is possible this is much higher in reality. Other observational studies have suggested an increased risk of repeat third or fourth degree tears in subsequent pregnancies of over two-fold to six-fold.[10–15,18,25] Contrastingly, others have reported that having an initial third or fourth degree tear was not associated with repeat third or fourth degree tear in the subsequent pregnancy[17,26] or a subsequent caesarean section.[18,26] An observational study found that women with a prior third or fourth degree tear whose second birth was an operative vaginal birth had a significantly higher risk of repeat third or fourth degree tear.[18] Consequently it remains difficult to counsel women on future mode of delivery as our findings would suggest that the vast majority of women over the last two decades in Scotland have opted for planned caesarean section following initial OASIS injury. A randomised controlled trial would be the ideal methodology to determine the risk of repeat third- or fourth-degree tear in asymptomatic women after an initial third- or fourth-degree tear, but the ethical implications of such a study are controversial.

A systematic review and meta-analysis of observational studies[27] found that there was no difference in de novo or worsening faecal incontinence in women with an initial third or fourth degree tear who had second vaginal births versus planned caesarean sections. Another study found no decrease in quality of life reported after repeat vaginal birth in women with prior third or fourth degree tear who had no evidence of anal sphincter incompetence.[28] This would suggest that vaginal birth is a viable option after an initial third or fourth degree tear in women with no lasting sphincter damage. Contrastingly, other studies report women with an initial third or fourth degree tear and a subsequent vaginal birth risk worsening anal[29] or faecal incontinence.[5] The Royal College of Obstetricians and Gynaecologists (RCOG) guideline[3] suggests women who are symptomatic should be counselled regarding the option of planned caesarean section. Our findings suggest that many women may be opting for caesarean section and the reasons for this need to be explored. It could be assumed that those with a more severe injury would opt for a caesarean section and those with 3A or 3B tears would be more likely to opt for vaginal birth. Furthermore, women who are symptomatic after an initial tear may be more likely to have a subsequent caesarean section, though evidence for this is limited[27] and needs to be explored further.

In this population, women with an initial third- or fourth-degree tear were twelve times more likely to have an elective caesarean section in the second pregnancy. Edozien et al[25] report an 18-fold increase in odds of planned caesarean section for women with an initial third or fourth degree in England from 2004 until 2012. This trend is likely to have significant financial implications given the increase to the caesarean section rate, however there are no published economic evaluations to determine the cost-effectiveness of planned caesarean section versus planned vaginal birth for women with third- or fourth-degree tears. It is possible that the cost of repeat third- or fourth-degree tears with or without chronic sequelae would be more expensive to health services. Minalgia et al[30] suggest the number needed to treat (NNT) was 5 caesarean sections to prevent 1 OASIS. McKenna et al[31] suggests that 2.3 caesarean sections would be needed to prevent one case of anal incontinence in women with previous third or fourth degree tear. Contrastingly, Elfaghi et al[12] suggest that the NNT was 23 caesarean sections.

Fourth degree tears have been associated with a higher risk of anal incontinence.[32] Second vaginal delivery was associated with increased anal incontinence if women had an initial fourth degree tear but not a third degree tear.[32] Elfaghi et al[12] report that the risk of repeat injury was increased six-fold for women with an initial fourth degree tear. More research is needed to differentiate the reproductive implications according to the severity of third- or fourth-degree tear.

Our findings suggest babies weighing ≥4500g and older mothers had an increased risk of repeat third- or fourth-degree tear. However due a small sample size it is possible this study is underpowered to detect a difference for the other risk factors studied. From the literature, forceps deliveries[10,11,15]; increased maternal age[12]; babies weighing ≥3500g[10]; babies weighing >5000g[11,12]; midline episiotomy[15]; large maternity hospitals[10]; were associated with repeat third or fourth degree tears. Epidural anaesthetic was found to be protective.[11] One study investigated third and fourth degree tears in an Italian population and found moderate / severe obesity was associated with such injuries.[33] However, we found no association with increasing BMI and the risk of repeat third or fourth degree tear. Interestingly, we found that more women with an initial third- or fourth-degree tear but no repeat third- or fourth-degree tear in the second vaginal birth had a forceps delivery in the second birth compared to cases (3.4% versus 2.7% respectively). However, this difference was not statistically significant. If sample size for the nested case-control study had been sufficiently large, it would have allowed for study of the association between instrumental delivery in both first and second births and the risk of repeat third and fourth degrees tears. A larger sample size would also have enabled evaluation of the concomitance of episiotomy with instrumental births.

Episiotomy use[34] and specifically restrictive episiotomy use[35], as well as episiotomy use at vacuum deliveries[36,37] have been associated with a reduction in risk of third and fourth degree tears in observational studies. However, evidence is conflicting with others reporting episiotomy has no effect on risk of third or fourth degree tear[33,38] or that episiotomy increases the risk of risk of third or fourth degree tear.[39] Episiotomy increased the risk of third or fourth degree tear when performed at instrumental deliveries in an observational study but was protective at spontaneous vaginal births,[40] which suggests that the increased risk could be secondary to instrumental vaginal birth as opposed to episiotomy. An observational study[41] suggests that with declining episiotomy use came a higher rate of anal sphincter injuries in a Finnish population, though the findings suggest that episiotomy did not protect women who were already at high risk. Prophylactic episiotomy use is not currently recommended by the RCOG.[3] The RCOG recommends use of episiotomy only when clinically indicated.[3] However, our results suggest that women with a previous history of third or fourth degree tear are significantly more likely to have an episiotomy in a second vaginal birth. This may reflect patient request. Alternatively, this may represent a belief by midwifery and medical staff that there is a beneficial effect. Robust research such as a randomised controlled trial to determine whether or not a prophylactic episiotomy is effective at preventing third- or fourth-degree tears in a second vaginal birth for women with an initial injury is needed. Furthermore, angled and specially designed episiotomy scissors[42] need to be assessed in a randomised controlled trial to determine their effectiveness at reducing the risk of both initial and repeat third and fourth degree tears. There was no association found with episiotomy use in second births which resulted in repeat third- or fourth-degree tears in this study, however these results need to be interpreted with caution as there is a risk of a type two error.

Third- and fourth-degree tears are a significant obstetric problem. Techniques need to be developed to prevent de novo anal sphincter injury. A Cochrane review found that warm compresses applied to the perineum during labour significantly reduced severe perineal trauma,[43] however there are no other preventative measures known. Endo-anal ultrasound studies[44] have shown that not all injuries to the anal sphincter are identified by clinicians. Therefore, focus should be on methods, whether using ultrasound or adequate training, to ensure appropriate identification of third- and fourth-degree tears. Predicting women at higher risk of third or fourth degree tears has been studied in the past.[33,45] An effective prediction model to identify women at higher risk of first third or fourth degree tear and to predict those with an initial injury who are at increased risk for a repeat injury would be ideal to help aid decision-making for women. Furthermore, qualitative research is needed to explore women’s views after enduring a third- or fourth-degree tear and to determine their priorities for subsequent births.

## Conclusions

Third- and fourth-degree tears are an increasing obstetric issue and can affect subsequent mode of delivery. Improving the prevention of these injuries needs to be prioritised.
